# Decoding the structure and translational promise of ROOL RNA nanocages

**DOI:** 10.1186/s43556-025-00315-1

**Published:** 2025-10-12

**Authors:** Hanqi Lou, Yuemin Ding, Shiwei Duan

**Affiliations:** Department of Clinical Medicine, School of Medicine, Hangzhou City University, Hangzhou, Zhejiang 310014 P.R. China

The study by Ling et al., recently accepted in Nature [1], represents a pioneering leap, providing the first atomic-resolution cryo-EM analysis of natural RNA nanocages assembled by the ROOL (rumen-originating, ornate, large) RNA family and bearing great importance for the subsequent development and functional application of ROOL RNA (Fig. 1). Long noncoding RNAs (lncRNAs) regulate key life processes, yet bacterial and bacteriophage lncRNAs (20 + classes) remain poorly understood, with high-resolution studies hindered by size and complexity.

**Fig. 1:**
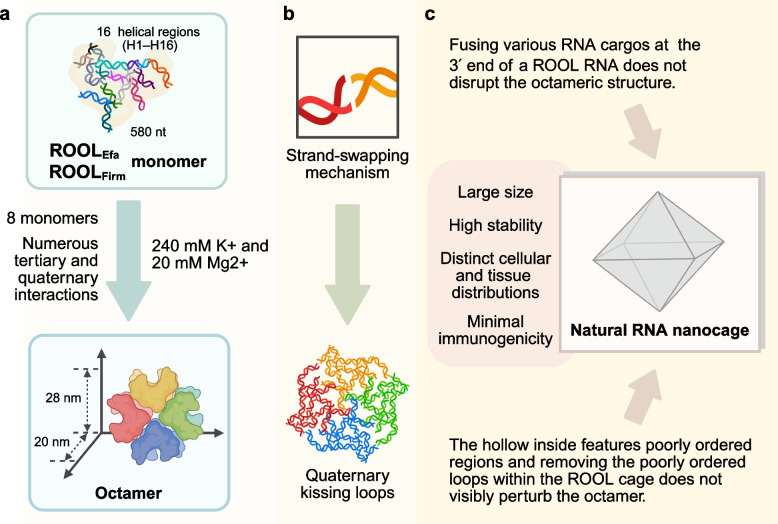
Eight ROOL monomers constitute the native ROOL RNA nanocage. **a **Eight ROOL monomers assemble into an octamer through extensive tertiary and quaternary interactions, forming a structure with a 28 nm diameter, 20 nm axial length, and disordered regions within its internal cavity. Octamer formation is favored with 240 mM K^+^ and 20 mM Mg^2+^. **b** Assembly of this nanocage occurs via a strand-exchange mechanism that generates a quaternary kissing loop, while disordered structures remain within the internal cavity. **c** ROOL haves great potential as a stable platform for the delivery of RNA or other cargo delivery in research and therapeutics. This naturally derived RNA nanocage stands out for its large size, high stability, unique cellular/tissue distribution, and low immunogenicity. Created in BioRender. L, H. (2025) https://BioRender.com/80txi3c. mM, Millimolar, nt, nucleotide, ROOL, rumen-originating, ornate, large

Ling et al.’s study delivers several key achievements. The authors resolved the structures of two bacterial ROOL RNAs—ROOL_Efa_ from Enterococcus faecalis and ROOL_Firm_ from Firmicutes bacterium CAG:227—to near-atomic resolutions (~ 2.9 Å for the octamer, ~ 3.2 Å for the monomer). Each RNA monomer comprises 16 helical regions (H1–H16), arranged in a slightly bent conformation stabilized by intricate tertiary interactions such as kissing loops, base stacking, and the newly coined "A-minor staples," which are triple-strand A-minor interactions that hold together structural elements like H4, H9, and H10. These monomers assemble into a symmetric octameric nanocage with a diameter of 28 nm and an axial length of 20 nm. The interior of the nanocage is hollow, with flexible regions, suggesting a potential for cargo encapsulation.

Importantly, their study unravels a strand-swapping mechanism critical to nanocage assembly. Upon oligomerization, helix regions H8-H10 and H12 undergo spatial shifts of ~ 23 Å and ~ 41 Å, respectively, enabling the formation of intermolecular base-pairing with adjacent monomers. Mutagenesis targeting the H12 region validated the functional necessity of this rearrangement, as disrupting this flexibility impairs octamer formation. The authors also characterized the intermediate oligomeric states using mass photometry and cryo-EM, reinforcing the modular assembly nature of the nanocage.

Beyond its structural novelty, the ROOL RNA nanocage presents compelling potential as a modular nanocarrier. The authors demonstrated that fusing functional RNA cargos—such as a Mango-III aptamer, a suppressor tRNA precursor, or a primary microRNA—at the 3′ end of ROOL RNA does not compromise the formation or structural integrity of the octamer. These cargos are displayed radially, and deletion of disordered internal loops does not disrupt nanocage formation, highlighting the scaffold's robustness and adaptability for payload delivery. The large size, structural stability, and potential low immunogenicity of this naturally derived RNA nanocage underscore its promise for RNA-based therapeutic delivery.

Moreover, the ROOL nanocage structure opens exciting avenues for biosensor development. By serving as a signal scaffold, the nanocage could be modified to act as a responsive fluorescent or electrochemical sensor. For instance, fusing aptamers to the nanocage surface could allow for conformational changes upon target molecule binding, triggering optical or electrochemical signal output. The hollow cavity could be loaded with fluorescent dyes or quantum dots, whose release or spectral shift would be modulated by the target-induced permeability changes. Additionally, the strand-exchange mechanism intrinsic to octamer assembly can function as a molecular switch: binding of target molecules may disrupt or enhance cage assembly, thereby modulating signals that can be detected via size-exclusion chromatography or dynamic light scattering. Such features could also be exploited for signal amplification in multiplexed biosensing platforms [2].

In the delivery of microRNAs (miRNAs), RNA nanocages demonstrate superior performance compared to conventional delivery systems, including lipid nanoparticles (LNPs) and viral vectors. Unlike LNPs, which frequently elicit immunogenic responses owing to their synthetic lipid components and are plagued by "accelerated blood clearance" phenomena, RNA nanocages—particularly natural variants such as ROOL—exhibit minimal immunogenicity and outstanding biocompatibility. Furthermore, they circumvent the safety hazards of insertional mutagenesis inherent to viral vectors. Notably, RNA nanocages support modular cargo loading and site-specific targeting modifications, while their structural conformation can be dynamically modulated by ionic conditions, thereby facilitating efficient intracellular cargo release. Collectively, these advantageous characteristics markedly augment the translational potential of RNA nanocages in miRNA-based therapeutic strategies and precision biomedicine applications.

Compared with previously reported synthetic RNA nanostructures, ROOL RNA, as a naturally occurring lncRNA, can achieve efficient folding and stable assembly without the need for artificial sequence design or structural modification, significantly simplifying the preparation process and improving structural reliability. Furthermore, the size of synthetic RNA nanostructures is often limited, whereas the nanocage self-assembled by ROOL has a diameter of up to 28 nm, and its internal cavity is sufficiently large to accommodate natural biological macromolecules such as tRNA and mRNA, endowing it with far superior functional loading potential than the former. ROOL RNA also exhibits prominent advantages over other natural RNA nanostructures: most natural RNA nanostructures have specific functions and low structural conservation, while ROOL not only maintains high quaternary structure conservation across different species but also possesses the property of modular modification. These unique attributes collectively highlight the great application prospects of ROOL RNA in fields such as nanocarriers and biosensors.

Despite these advances, several limitations and challenges remain. The linear relationship between nanocage structural dynamics and signal transduction remains to be firmly established, a key issue for quantitative biosensing. In vivo application also raises concerns regarding the clearance mechanisms and immunogenicity of ROOL RNAs in mammalian systems. Although these RNAs are naturally derived from bacteria, their introduction into mammals may provoke immune responses, necessitating chemical modifications or structural reengineering [3]. Further, the functional stability of these nanocages in complex biological environments must be rigorously assessed.

Looking forward, future research should delve deeper into the regulation and dynamics of ROOL nanocage assembly. Detailed molecular dynamics simulations and mutational analyses can further elucidate how environmental conditions and sequence variations influence their structural equilibrium. Application-wise, ROOL-based carriers can be tailored for targeted gene or drug delivery by decorating their surfaces with disease-specific ligands—particularly for oncology or virology contexts [4]. In the field of diagnostics, coupling these nanocages with nanophotonic and single-molecule detection technologies could significantly enhance the sensitivity and specificity of biomarker detection [5]. Long-term in vivo safety and efficacy studies will be critical to validate their translational potential.

In summary, their study provides a groundbreaking structural and functional dissection of natural RNA nanocages and marks a transformative step in bacterial lncRNA research and RNA nanotechnology. The architectural elegance, modularity, and application versatility of ROOL RNAs herald new frontiers in therapeutic delivery systems and biosensing platforms. Though challenges remain, the roadmap laid by Ling et al. sets the stage for a wide range of biotechnological and clinical innovations grounded in natural RNA engineering.

## Data Availability

Not applicable.

## Abbreviations

lncRNAs: Long noncoding RNAs
LNPs: Lipid nanoparticles
miRNAs: MicroRNAs
ROOL: Rumen-originating, ornate, large
